# Supplementary material for: Family caregivers’ emotional and communication needs in Canadian pediatric emergency departments
Source: PLoS One. 2023 Nov 22;18(11):e0294597. doi: 10.1371/journal.pone.0294597 (PMC10664925; doi:10.1371/journal.pone.0294597)
Supplement: S1 Table — (DOCX) [file pone.0294597.s002.docx]

**Supplementary Table 1: ED visit details**

| **Mode of arrival (n=1953)** | | |
| --- | --- | --- |
| Private vehicle | | 1788 (91.6) |
| Ambulance | | 97 (5.0) |
| Walk-in | | 55 (2.8) |
| Other | | 13 (0.7) |
| **Reason for visit (n=1954)** | | |
| I thought we needed ED treatment | | 1099 (56.2) |
| Sent in by a doctor | | 405 (20.7) |
| Child’s own doctor not available | | 215 (11.0) |
| Telephone advice line said to come in | | 126 (6.4) |
| No family doctor/primary care doctor | | 33 (1.7) |
| Follow up, PRN, caregiver discretion | | 17 (0.9) |
| Out of home emergency, third party decision | | 12 (0.6) |
| Other | | 47 (2.4) |
| **Time to be seen by a physician (minutes) (n=1939)** | | |
| Mean (SD) | | 101.8 (82.5) |
| Median (IQR) | | 82.0 (39.0-144.0) |
| **Length of Stay (LOS) (hours) (n=1967)** | | |
| Mean (SD) | | 5.5 (6.8) |
| Median (IQR) | | 3.9 (2.6-6.0) |
| **Interventions Received (n=1550)*** | | |
| Oral medications | | 912 (58.9) |
| Labs/bloodwork | | 755 (48.7) |
| X-ray/Medical Imaging | | 624 (40.3) |
| IV insertion | | 415 (26.8) |
| Consultation with another specialty | | 322 (20.8) |
| IV medications | | 252 (16.3) |
| Inhaled medications | | 163 (10.5) |
| Medication (another route) | | 115 (7.4) |
| In ED procedure (laceration repair, lumbar puncture) | | 77 (5.0) |
| ECG | | 57 (3.7) |
| Cast application/Other immobilization | | 36 (2.3) |
| Procedural sedation | | 21 (1.4) |
| Other | | 21 (1.4) |
| **Final diagnosis (system affected) (n=2005)** | |  |
| Respiratory | | 446 (22.2) |
| Gastrointestinal | | 441 (22.0) |
| Musculoskeletal | | 257 (12.8) |
| Head and neck | | 193 (9.6) |
| Skin issues | | 174 (8.7) |
| Fever and Infection NOS | | 169 (8.4) |
| Neurologic | | 132 (6.6) |
| Genito-urinary | | 123 (6.1) |
| Laceration and Trauma NOS | | 76 (3.8) |
| Cardiovascular | | 37 (1.8) |
| Allergy/Immunology | | 22 (1.1) |
| Hematology/Oncology | | 18 (0.9) |
| Other | | 56 (2.8) |
| **Previous Hospitalizations (n=1970)** | | |
| 0 | 1336 (67.8) | |
| 1-5 | 549 (27.9) | |
| 6-10 | 27 (1.4) | |
| > 10 | 58 (2.9) | |

*multiple interventions for each child possible; n=455 received no interventions
