# Supplementary material for: Family caregivers’ emotional and communication needs in Canadian pediatric emergency departments
Source: PLoS One. 2023 Nov 22;18(11):e0294597. doi: 10.1371/journal.pone.0294597 (PMC10664925; doi:10.1371/journal.pone.0294597)
Supplement: S2 Table — (DOCX) [file pone.0294597.s003.docx]

**Supplementary Table 2: Univariable logistic regression model for likelihood of emotional needs being met**

| **Variable** | **Odds Ratio (95% CI)** | **p-value** |
| --- | --- | --- |
| Previous visits to ED |  | 0.54 |
| 6-10 vs 1-5 | 1.16 (0.83, 1.63) | 0.39 |
| >10 vs 1-5 | 0.99 (0.66, 1.47) | 0.95 |
| Never vs 1-5 | 1.22 (0.90, 1.67) | 0.20 |
| CTAS (4 categories) |  | **0.02** |
| 3 – Urgent vs 1+2 | **0.62 (0.45, 0.85)** | **0.003** |
| 4 – Semi urgent vs 1+2 | **0.63 (0.44, 0.90)** | **0.01** |
| 5 – Non urgent vs 1 + 2 | **0.50 (0.27, 0.93)** | **0.03** |
| CTAS (4 categories), continuous | **0.83 (0.72, 0.96)** | **0.01** |
| Chronic Illness |  | 0.63 |
| Unsure vs No | 0.82 (0.54, 1.24) | 0.35 |
| Yes vs No | 1.00 (0.75, 1.34) | 0.98 |
| Relationship to child |  | **0.008** |
| Mother vs Father | **0.66 (0.49, 0.87)** | **0.004** |
| Other vs Father | 1.28 (0.42, 3.89) | 0.67 |
| Number of other kids |  | 0.15 |
| 1 vs 0 | 1.29 (0.97, 1.72) | 0.09 |
| 2 vs 0 | 0.97 (0.69, 1.38) | 0.87 |
| 3+ vs 0 | 0.96 (0.64, 1.45) | 0.85 |
| Did you feel that your child’s privacy was respected? | **2.17 (1.87, 2.51)** | **< 0.0001** |
| Did you wonder whether you should have come to the hospital sooner? | 0.94 (0.86, 1.03) | 0.16 |
| Did you feel scared during the ED visit? | **0.76 (0.69, 0.83)** | **< 0.0001** |
| Did the doctors, nurses, and other providers involve YOU in your child’s care? | **2.36 (2.06, 2.71)** | **< 0.0001** |
| How satisfactory were the updates to you about your child’s care in the ED? | **2.13 (1.91, 2.38)** | **< 0.0001** |
| Did the emergency staff answer your questions and concerns? | **2.85 (2.47, 3.29)** | **< 0.0001** |
| Child’s Age | 1.00 (0.98, 1.02) | 0.80 |
| Caregiver’s Age | 1.01 (0.99, 1.02) | 0.33 |
| STAI score | **0.95 (0.94, 0.96)** | **< 0.0001** |
| NVS score | **1.09 (1.03, 1.17)** | **0.005** |

*table presents estimates for the odds ratio of caregiver’s emotional needs met (answers 4+5)
