# Supplementary material for: Family caregivers’ emotional and communication needs in Canadian pediatric emergency departments
Source: PLoS One. 2023 Nov 22;18(11):e0294597. doi: 10.1371/journal.pone.0294597 (PMC10664925; doi:10.1371/journal.pone.0294597)
Supplement: S3 Table — (DOCX) [file pone.0294597.s004.docx]

**Supplementary Table 3: Univariable logistic regression model for caregiver comfort in caring for their child’s illness at home**

| **Variable** | **Odds Ratio (95% CI)** | **p-value** |
| --- | --- | --- |
| Previous visits to the ED |  | 0.85 |
| 6-10 vs 1-5 | 1.03 (0.68, 1.56) | 0.88 |
| >10 vs 1-5 | 1.12 (0.65, 1.92) | 0.68 |
| Never vs 1-5 | 1.18 (0.80, 1.74) | 0.40 |
| CTAS (4 categories) |  | 0.27 |
| 3 – Urgent vs 1+2 | 0.73 (0.49, 1.09) | 0.12 |
| 4 – Semi urgent vs 1+2 | 0.76 (0.49, 1.19) | 0.23 |
| 5 – Non urgent vs 1 + 2 | 1.32 (0.53, 3.32) | 0.55 |
| Chronic Illness |  | 0.87 |
| Unsure vs No | 0.93 (0.55, 1.58) | 0.80 |
| Yes vs No | 1.08 (0.75, 1.56) | 0.67 |
| Previous Hospitalizations |  | 0.56 |
| 6-10 vs 1-5 | 1.55 (0.34, 6.99) | 0.57 |
| >10 vs 1-5 | 1.99 (0.69, 5.80) | 0.21 |
| None vs 1-5 | 0.98 (0.72, 1.35) | 0.92 |
| Relationship to the child |  |  |
| Mother vs father | **0.67 (0.47, 0.96)** | **0.03** |
| Number of other kids |  | 0.08 |
| 1 vs 0 | 1.15 (0.80, 1.64) | 0.45 |
| 2 vs 0 | 1.30 (0.82, 2.06) | 0.27 |
| 3+ vs 0 | 0.69 (0.43, 1.11) | 0.12 |
| Did you wonder whether you should have come to the hospital sooner? | **0.85 (0.76, 0.94)** | **0.001** |
| Did you feel scared during the ED visit? | **0.63 (0.57, 0.71)** | **< 0.0001** |
| Did the doctors, nurses, and other providers involve YOU in your child’s care? | **2.03 (1.76, 2.35)** | **< 0.0001** |
| How satisfactory were the updates to you about your child’s care in the ED? | **2.07 (1.83, 2.35)** | **< 0.0001** |
| Did the emergency staff answer your questions and concerns | **2.68 (2.31, 3.11)** | **< 0.0001** |
| Child age | 0.98 (0.96, 1.01) | 0.20 |
| Caregiver age | 1.01 (0.99, 1.03) | 0.38 |
| STAI score | **0.94 (0.93, 0.95)** | **< 0.0001** |
| NVS score | **1.10 (1.02, 1.19)** | **0.01** |

*table presents estimates for the odds ratio of caregivers comfortable to care for the child at home (answers 4+5)
