# Supplementary material for: Family caregivers’ emotional and communication needs in Canadian pediatric emergency departments
Source: PLoS One. 2023 Nov 22;18(11):e0294597. doi: 10.1371/journal.pone.0294597 (PMC10664925; doi:10.1371/journal.pone.0294597)
Supplement: Attachment — Submitted filename: TABLE - Ali - PLOS ONE 28oct23.docx [file pone.0294597.s006.docx]

**Response to Reviewers: PLOS ONE (October 2023)**

*“Family caregivers’ emotional and communication needs in Canadian pediatric emergency departments”*

| **Reviewer** | **Section** | **Comment** | **Response** |
| --- | --- | --- | --- |
|  | General | Please ensure that your manuscript meets PLOS ONE's style requirements, including those for file naming. The PLOS ONE style templates can be found at:  <https://journals.plos.org/plosone/s/file?id=wjVg/PLOSOne_formatting_sample_main_body.pdf> and  <https://journals.plos.org/plosone/s/file?id=ba62/PLOSOne_formatting_sample_title_authors_affiliations.pdf> | Thank you. We have relabeled Supplementary files as requested and confirmed that the manuscript meets PLOS ONE’s style requirements. |
|  |  | Thank you for stating in your Funding Statement:  "This study was supported by the Women and Children’s Health Research Institute (WCHRI) and the Clinical/Community Research Integration Support Program (CRISP) grant, secured by Dr. Shannon Scott and Dr. Samina Ali (2018-2022)."  Please provide an amended statement that declares *all* the funding or sources of support (whether external or internal to your organization) received during this study, as detailed online in our guide for authors at <http://journals.plos.org/plosone/s/submit-now>.  Please also include the statement “There was no additional external funding received for this study.” in your updated Funding Statement.  Please include your amended Funding Statement within your cover letter. We will change the online submission form on your behalf. | Thank you. This has been modified in the cover letter to state:  “This work was supported by the Women and Children’s Health Research Institute *via both a Clinical/Community Research Integration Support Program grant secured by Drs. Samina Ali and Shannon Scott (2018-2022) and a trainee grant* *secured by Dr. Claudia Maki (2022-2023)* through a generous donation from the Stollery Children’s Hospital Foundation. *The funders of the study had no role in study design, data collection, data analysis, data interpretation, or writing of the report*. *There was no additional external funding received for this study*.”  This now represents all funding for this project. |
|  |  | In your Data Availability statement, you have not specified where the minimal data set underlying the results described in your manuscript can be found. PLOS defines a study's minimal data set as the underlying data used to reach the conclusions drawn in the manuscript and any additional data required to replicate the reported study findings in their entirety. All PLOS journals require that the minimal data set be made fully available. For more information about our data policy, please see <http://journals.plos.org/plosone/s/data-availability>.  Upon re-submitting your revised manuscript, please upload your study’s minimal underlying data set as either Supporting Information files or to a stable, public repository and include the relevant URLs, DOIs, or accession numbers within your revised cover letter. For a list of acceptable repositories, please see <http://journals.plos.org/plosone/s/data-availability#loc-recommended-repositories>. Any potentially identifying patient information must be fully anonymized.  Important: If there are ethical or legal restrictions to sharing your data publicly, please explain these restrictions in detail. Please see our guidelines for more information on what we consider unacceptable restrictions to publicly sharing data: <http://journals.plos.org/plosone/s/data-availability#loc-unacceptable-data-access-restrictions>. Note that it is not acceptable for the authors to be the sole named individuals responsible for ensuring data access.  We will update your Data Availability statement to reflect the information you provide in your cover letter. | The authors appreciate this policy very much. Moving forward, with our consent forms, we will indicate that this is expected for participants. For this study, which was approved by our ethics boards a number of years ago, we did not specify that de-identified data were to be stored in a publicly available repository. As such, we suggest the following be stated for this manuscript: “Data cannot be shared publicly because of consent and confidentiality reasons. Data are available from the Program Manager, Pediatric Emergency: Advancing Knowledge team Mrs. Pat Candelaria (stacruz@ualberta.ca) or the corresponding author Dr. Samina Ali (sali@ualberta.ca) for researchers who meet the criteria for access to confidential data.” |
|  |  | Please note that in order to use the direct billing option the corresponding author must be affiliated with the chosen institute. Please either amend your manuscript to change the affiliation or corresponding author, or email us at plosone@plos.org with a request to remove this option. | The corresponding author, Dr. Samina Ali, has a primary affiliation with University of Alberta. Per your website, direct billing for PLOS ONE is available to this institution. <https://plos.org/resources/for-institutions/institutional-account-participants/>  Please advise if any further action is required. |
|  |  | One of the noted authors is a group or consortium the Pediatric Emergency Research Canada Family Needs Study Group. In addition to naming the author group, please list the individual authors and affiliations within this group in the acknowledgments section of your manuscript. | Thank you for this reminder. This has been done in the Acknowledgments section, already. |
|  |  | Please also clearly indicate a lead author for this group along with a contact email address. | We have added the following to the Acknowledgements section: *“Dr. Samina Ali (*[*Sali@ualberta.ca*](mailto:Sali@ualberta.ca)*) is the lead author for this group.”* |
|  |  | We are unable to open your Supporting Information file Appendix 1 Family Needs Survey 6jun2023.pdf. Please kindly revise as necessary and re-upload. | We have attached a new version of this document, entitled “Family Needs Survey Tool 23oct23.pdf”. |
|  |  | Please review your reference list to ensure that it is complete and correct. If you have cited papers that have been retracted, please include the rationale for doing so in the manuscript text, or remove these references and replace them with relevant current references.  Any changes to the reference list should be mentioned in the rebuttal letter that accompanies your revised manuscript. If you need to cite a retracted article, indicate the article’s retracted status in the References list and also include a citation and full reference for the retraction notice. | To our knowledge, all references are current and accurately represented.  We have added the following new reference:  Why Parents Seek Care for Acute Illness in the Clinic or the ED: The Role of Health Literacy.  May M, Brousseau DC, Nelson DA, Flynn KE, Wolf MS, Lepley B, Morrison AK.  Acad Pediatr. 2018 Apr;18(3):289-296. doi: 10.1016/j.acap.2017.06.010. Epub 2017 Jun 16.  PMID: 28625711 |
| 1 | General | Thank you very much for inviting me to read this good manuscript that highlights the importance of doctors and nurses in emergency units and the impact this has on child care providers. I will allow myself to make the following observations to the manuscript to raise its academic quality: | Thank you for your time and efforts in reviewing this manuscript. |
|  | Methods | The described methodology of the manuscript presents several strengths and noteworthy considerations. The study's strengths include its comprehensive approach to data collection and analysis. The sub-study employs a descriptive cross-sectional survey coupled with medical record review, providing a multi-faceted view of caregivers' experiences in pediatric emergency departments (EDs). The utilization of a convenience sample from ten Canadian pediatric EDs enhances the study's external validity, making the findings more generalizable. The rigorous development and validation process of the survey tools, involving expert panel input, pre-testing, pilot testing, and sensibility testing, contribute to the robustness of the measures used to assess caregiver needs and experiences. | Thank you for noting this. We aimed to have a rigorous, patient-included methodologic approach to this study. |
|  |  | However, there are also potential challenges and limitations to consider. The use of a convenience sample might introduce selection bias, as participants may not fully represent the diversity of all families visiting pediatric EDs. | We wholly agree with you. While we cannot change the methods at this time, we have added the following (represented in italicized script) to the Limitations section:  “The research assistants administered the surveys during daytime and evening hours, *as a convenience sample*, perhaps not capturing the unique needs of caregivers during the overnight period. This study focuses on caregiver experiences only, not capturing the emotional needs of the full family (e.g., child, other caregivers). The survey *also* excluded caregivers whose child was medically unstable, therefore we may not have captured the emotional needs of caregivers with the sickest children. *As such, this study may not fully represent the diversity of all families visiting pediatric EDs.”* |
|  |  | Despite efforts to ensure robust survey tools, self-reporting may introduce recall or response biases. I recommend that the authors describe in more detail how they did to reduce the risk of bias in the study. | The following was added to the Limitations Section: *“Despite our efforts to ensure robust survey tools, self-reporting may introduce recall or response biases.* *We attempted to mitigate this through careful selection of research questions, the administration of two surveys (one during the visit and a second one in follow-up), choosing an appropriate data collection method for the population (iPad or email), and collecting the data prospectively.”* |
| 2 | General | A very well prepared manuscript and clearly laid out methodology. | Thank you for noting this. We aimed to have a rigorous, patient-included methodologic approach to this study. |
|  | Abstract | Note that the objectives in the abstract are not in alignment with the objectives in the article. | *Apologies for this. In an attempt to achieve brevity in the abstract, the objectives were not clearly represented. We have now included the objectives from the manuscript, verbatim, so they match exactly.*  *“To describe the extent to which caregivers’ emotional and communication needs were met during pediatric emergency department (PED) visits. Secondary objectives included describing the association of caregiver emotional needs, satisfaction with care, and comfort in caring for their child’s illness at the time of discharge with demographic characteristics, caregiver experiences, and ED visit details.”* |
|  |  | Would you say that the findings presented in this article related the findings to the demographic characteristics? Perhaps rework to be in line with the objective presented in the article. | Due to an editing error on our part, we realized upon review that the objectives did not include the word ‘demographics’. This has been rectified, and the objectives are now as follows:  “To describe the extent to which caregivers’ emotional and communication needs were met during pediatric emergency department (PED) visits. Secondary objectives included describing the association of caregiver emotional needs, satisfaction with care, and comfort in caring for their child’s illness at the time of discharge with *demographic characteristics*, caregiver experiences, and ED visit details.”  We explored demographic characteristics, as they relate to caregiver emotional and communication needs, as demographic variables including caregiver age, child age, relationship to child, and number of other children we included in the univariable regression modeling (see S2 and S3 Files); ultimately, however, they were not significant enough to be included in the final multivariable model.  To make this more clear, we have added the following to the Analysis section, to indicate explicitly that demographic variables were explored through modeling in this study:  “Multivariable logistic regression was used for the outcomes to ascertain effects of specific independent variables (e.g., triage category, health literacy, state anxiety, *caregiver age, child age, number of other children*) on caregivers’ reported emotional and communication needs and their satisfaction.”  Due to space constraints in the brief abstract, we are not able to provide all univariable results, but have summarized the multivariable results, only, instead. |
|  | Introduction | Consider including a brief description of the term "caregiver satisfaction". This is obviously similar to but not the same as patient satisfaction which is not applicable to this study. | We have added the following to the Introduction:  “*Patient and caregiver satisfaction are intimately related, with caregiver satisfaction being dependent on the quality of interpersonal interaction, communication, empathy, and compassion demonstrated, and influencing the child’s experience in the pediatric setting*.” |
|  |  | The term "team" is utilised quite frequently. And in some instances is referred to as the ED team. It would be helpful to the reader to indicate early on who is being referred to when the term "team" is used. In some EDs there may be a designated nurse who would be a "constant" throughout the caregiver's time in ED. | We have added ‘*health care’* in front of all instances where we are referring to the clinical team and ‘*research*’ in front of all instances where we are referring to our research team.  Further, we have added *“(i.e., bedside nurse, physician, child life specialist, social worker, etc)”* at the first instance of ‘health care team’ being mentioned, in order to clarify who we are referring to. For the hospitals involved in this study, their care team can include others besides only the bedside nurse, so we attempted to capture this with the broader term ‘health care team, rather than referring only to ‘bedside nurse’, for example. |
|  |  | First and only time the PICU acronym is used. Recommend writing in full. Just a query if this should be an ED setting? | PICU has been changed to ‘*pediatric intensive care* setting’. As most work in the emotional needs of families has been done in the pediatric intensive care setting, we chose to include this one reference to a recently completed systematic review of the topic. The remainder of our references are essentially limited to the emergency setting. |
|  |  | **Page 5; Line 7:** What is meant by "Characterizing the emotional and communication needs...."? | We have changed this to “*Better understanding* the emotional and communication needs of caregivers in the ED could identify opportunities to be more responsive to families’ needs.”  We trust that this makes the intention of the sentence clear. |
|  |  | **Page 5; Line 13:** The term "satisfaction" is presented alone. It needs qualification. Satisfaction with what? | We have added the following to made this objectives more clear:  “Secondary objectives included describing the association of caregiver emotional needs, satisfaction *with care*, and comfort in caring for their child’s illness at the time of discharge with visit characteristics (e.g., triage category, previous visits to the ED), caregiver experiences, and ED visit details. |
|  |  | **Page 5; Line 14:** The term "visit characteristics" also needs elaborating upon | Thank you. due to an editing error on our part, we realized upon review that the objectives did not include the phrase ‘demographic characteristics’ and was accidentally typed as ‘visit characteristics’. This has been rectified, and the objectives are now as follows:  “To describe the extent to which caregivers’ emotional and communication needs were met during pediatric emergency department (PED) visits. Secondary objectives included describing the association of caregiver emotional needs, satisfaction with care, and comfort in caring for their child’s illness at the time of discharge with *demographic characteristics*, caregiver experiences, and ED visit details.” |
|  | Methods | **Page 7; Line 13:** Was the word "permuted" intentional? If yes, maybe change from "missing responses" to "missed responses". | This has been adjusted to ‘*missed responses’*, as suggested. |
|  |  | **Page 8; Line 16:** Clarify: satisfaction with what? | As with the study objectives, this has now been updated to state:  “Secondary outcomes included describing the association of emotional needs, satisfaction *with care*, and caregiver comfort in caring for their child’s illness at the time of discharge, with demographic characteristics, caregiver experiences, and ED visit details.” |
|  | Discussion | **Page 18; Line 15:** Is it to improve the overall patient experience or the overall caregiver experience? - the focus of this study being on the caregiver. | We have modified this to state:  “Our study demonstrates the importance of all healthcare professionals working together to meet the emotional needs of caregivers in the ED to improve the overall *family* experience.”  We have done so as the child and caregiver health care experiences are intimately tied together, and health care teams value both. |
|  |  | **Page 18; Line 20:** Kindly provide some literature to show whether existing data supports/contradicts these findings. | We have added the following, with 2 references, to make this Discussion more robust. Thank you for the suggestion.  *“Privacy and caregiver gender have not been previously explored in relation to emotional needs in the ED. However, prior study has demonstrated that families with low health literacy are more likely to overestimate severity of illness, seek care for lower acuity illness, and desire supportive care (including emotional needs) sooner though the ED.”* |
|  |  | **Page 19; Line 1-4:** As above, kindly show how existing research supports/contradicts these findings. | We have added the following, with a new reference:  “*We know that diagnostic uncertainty is associated with higher return visits to the ED for children and their caregivers; while the connection to emotional needs has not been explicitly made prior to our current study, it stands to reason that feeling unsure and unsafe (i.e., unmet emotional needs) likely has a role in this return visit behaviour. This needs to be further explored through qualitative inquiry to better understand the relationship between emotional needs and comfort caring for a child after an ED visit.*”  Please note that a fulsome discussion of the impact of caregiver anxiety follows shortly after this section. We felt it was important enough to merit a separate paragraph, rather than including it here. |
|  |  | **Page 19; Line 20:** Kindly elaborate on/qualify the term satisfaction, i.e. satisfaction with what? | We have clarified as follows:  “These findings underscore the importance of caregiver involvement in their child’s care and adequate communication by ED staff as critical driving factors in improving emotional and communication needs and satisfaction *with care* for caregivers in the ED.” |
|  |  | **Page 20; Line 7-9:** Kindly note that anxiety levels and emotional needs are not necessarily the same thing. One may have one's emotional needs met, but anxiety is still present. Rather find another study that refers to emotional needs. However, it is an interesting outcome. | Thank you for this important distinction. We have reworded this sentence to reflect that anxiety and emotional needs are separate concepts that are inter-related, but not the same thing.  *“It* has been previously shown that caregiver anxiety levels are *higher with greater acuity of their child’s illness;[*2] our study found higher acuity to be associated with caregiver emotional needs more likely to be met.” |
|  |  | **Page 22; Line 5:** Increase satisfaction with what? | We have reworded as follows, to address this:  “This anxiety can be greatly impacted by PFCC interventions to reduce stress, anxiety, *and* depression, increase *satisfaction with care,* and improve relationships with health care providers.”  Of note, we have made this clarification throughout the paper, to address the reviewer’s concern. |
|  | Conclusion | It is recommended that the conclusion also includes the many positive outcomes of the findings. For instance, the majority of caregivers had their emotional needs met and the majority of caregivers had their communication needs met. And this is across Canada's pediatric EDs. This is a highly commendable outcome. | We appreciate this positive lens on our results. We have specifically chosen to point out the ‘deficits’ in order to use this as a springboard to suggest what can be done to further improve family experiences. While we agree that 85% of caregivers reporting their communication needs as being met may seem excellent, our study still reports that 1/3 caregivers have unmet emotional needs and 1/6 have unmet communication needs when they are leaving the ED. As a large team of ED care providers, researchers, and people with lived experience, our team wishes to call the EDs to action to do better than this. It is not a judgement, but a reminder that we have capacity and resources to do better. |
|  |  | **Page 22; Conclusion, Line 4:** Does the word "influence" refer to influence for the better or worse? The apostrophe for caregivers should be after the "s" as caregivers'. And satisfaction with what? | We have altered the sentence as follows:  “These unmet needs *negatively* influence caregivers’ satisfaction *with care*, but even more importantly, their ability to comfortably care for their child’s illness at home.” |
|  |  | **Page 23; Conclusion; Last sentence:** The phrase "make an impact" - kindly clarify: Make an impact on what? | We have modified as follows:  “More complex systemic interventions (e.g., addressing of national ED overcrowding and staffing challenges) and public health initiatives to address caregiver health literacy and anxiety surrounding ED visits will also make *a positive* *impact on family ED experiences;* these will require concerted national efforts to rectify.” |
